# Mesenchymal stromal cells ameliorate acute allergic rhinitis in rats

**DOI:** 10.1002/cbf.3291

**Published:** 2017-09-20

**Authors:** Chunlei Li, Yanxia Fu, Yinyin Wang, Yanhua Kong, Mengdi Li, Danhui Ma, Wanli Zhai, Hao Wang, Yuting Lin, Sihan Liu, Fangli Ren, Jun Li, Yi Wang

**Affiliations:** ^1^ Graduate School of Beijing University of Chinese Medicine, Clinical Medical School of Beijing University of Chinese Medicine Beijing University of Chinese Medicine Beijing China; ^2^ National Clinical Research Center of Respiratory Diseases, Center for Respiratory Diseases China‐Japan Friendship Hospital, The 2nd Pulmonary Department of TCM, The Key Institute of State Administration of Traditional Chinese Medicine (Pneumonopathy Chronic Cough and Dyspnea), Beijing Key Laboratory (no BZ0321) China‐Japan Friendship Hospital Beijing China; ^3^ State Key Laboratory of Membrane Biology, School of Medicine Tsinghua University Beijing China; ^4^ Institute of Immunology, Medical School Third Military Medical University Chongqing China

**Keywords:** allergic rhinitis, antiinflammation, cytokine, mesenchymal stromal cells, umbilical cord

## Abstract

Mesenchymal stromal cells (MSCs) have been extensively investigated as a potential antiinflammatory treatment in many inflammatory‐related diseases; however, it remains unclear whether MSCs could be used to treat acute allergic rhinitis. A rat model of allergic rhinitis was treated with MSCs. The effect of MSCs on the inflammation of allergic rhinitis was evaluated by sneezing, nose rubbing, the pathology of the nasal mucosa, and the expression of interleukin 4, tumour necrosis factor alpha, and immunoglobulin E in the serum of rats. Also, the population of MSCs isolated from umbilical cords of humans was evaluated to determine if they could inhibit the symptoms and inflammation of acute allergic rhinitis in a rat model. We observed that this population of cells inhibited sneezing, nose rubbing, and changes in the pathology of the nasal mucosa. Intriguingly, we observed that MSCs reduced the expression of interleukin 4, tumour necrosis factor alpha, and immunoglobulin E in the serum. Furthermore, MSCs reduced the expression of histamine and the recruitment of macrophages in the nasal mucosa of allergic rhinitis rats. We reasoned that the effect of MSCs on allergic rhinitis might be through its regulation of the secretion of related cytokines from macrophages during the process of acute allergic rhinitis. This work suggested that MSCs from the umbilical cords of humans could be used as a positive clinical therapy for the human disease.

## 1 INTRODUCTION

Allergic rhinitis (AR) is a type of inflammation in the upper respiratory tract, and it occurs when the immune system overreacts to allergens in the air.^1^, ^2^ Allergic rhinitis is a common health problem in both children and adults. Nearly 30% of the world population suffers from this disease.^3^ The classical symptoms of AR include sneezing, rhinorrhoea, nasal itching, and congestion; multiple related symptoms may occur at the same time.^4^ Although the symptoms resemble those of the common cold, they often last for more than 2 weeks and typically do not include a fever.^5^ Patients who suffer from AR sustain a significant negative impact on their quality of life.^5^, ^6^

Allergic rhinitis is an immune disorder, and it is caused by hypersensitivity towards all kinds of allergens.^1^, ^7^ Recent studies have suggested that T helper type 2 (Th2) cells and mast cells play crucial roles in the pathogenesis and process of allergic reactions during AR.^8^ T helper type 2 cells mediate the activation and maintenance of the allergens and secrete a population of Th2 cytokines, such as interleukin 4 (IL‐4). Then, more T helper cells are recruited into the allergic response and promote the synthesis and secretion of immunoglobulin E (IgE) from B lymphocytes.^8^, ^9^ Once IgE binds to the high‐affinity receptor (FcεRI) on the surface of mast cells, special IgE responses to antigens are switched on.^10^ Subsequently, the binding of IgE‐FcεRI activates mast cells, which leads to a degranulation response and the secretion of allergic mediators, including inflammatory cytokines and histamine, and results in the infiltration of inflammatory cells, followed by acute or chronic inflammation of the nasal mucosa. Moreover, IL‐4 suppresses Th1 and Th17 cell responses through the upregulation of transcriptional repressors of interferon gamma (IFN‐γ) and IL17 that play important roles in the process of AR.^11^, ^12^ Thus, the regulation of the disordered inflammatory cytokine secretion could be a potential strategy to cure AR.

Human mesenchymal stromal cells (MSCs) are multilineage stem cells that can be isolated from bone marrow, umbilical cord, amniotic membrane, fat aspirate, and dental pulp.^13^ In recent years, this population of cells has been reported to inhibit inflammation as a promising therapeutic strategy to many inflammatory diseases and cancers^14^, ^15^, ^16^; however, no study has been conducted to reveal the efficacy and possible mechanisms of human MSCs in the treatment of AR.

In the present study, we isolated MSCs from human umbilical cord and aimed to evaluate their effect on allergic inflammatory responses in an ovalbumin (OVA)‐induced AR model in rats. Intriguingly, we observed that MSCs from human umbilical cord appear to have significant therapeutic efficacy in inhibiting the symptoms of sneezing and nose rubbing in acute AR rats. We further analysed that the anti‐AR effect of MSCs might be associated with the regulation of cytokine secretion from macrophages during the process of AR. Our study provides hope for the therapy of AR using MSCs from human umbilical cord.

## 2 MATERIALS AND METHODS

### 2.1 Cells

Mesenchymal stromal cells were isolated with high efficiency from umbilical cord under the agreement of informed consent in the hospital and expanded in vitro with Eagle's minimal essential medium (Gibco/BRL, USA), supplemented with 10% foetal bovine serum and 1 × l‐glutamine, according to the method reported by Izaskun Ferrin et al.^13^ The Institution Review Board of Tsinghua University approved the entirety of the project and found it concurrent with all related protocols (no: 20170013).

### 2.2 Rats

A total of 60 male Sprague‐Dawley rats (160‐180 g) were purchased from Vital River Laboratories (Beijing, China). The rats were maintained in the Animal Resource Facility at Tsinghua University, fed with standard laboratory chow and water ad libitum, and maintained under a 12 hour dark/light cycle. The animal experiment was conducted with approval from and under the supervision of the Laboratory Animal Welfare and Ethics Committee of the Institute of Materia Medica, Chinese Academy of Medical Sciences (no 16‐CZJ1). Animal surgeries were performed under isoflurane anaesthesia with minimal suffering.

### 2.3 Ovalbumin‐induced allergic rhinitis rat models

The rats were sensitized by OVA by using a standard protocol. Briefly, OVA and aluminium hydroxide gel diluted in normal saline (1 mg/mL + 30 mg/mL + 1 mL) were used to sensitize the rats 3 times via injection in 5 sites (double back toes, bilateral inguinal subcutaneous region, and abdominal cavity, with 0.2 mL for each site) on days 1, 5, and 10. The same amount of normal saline was injected into the 5 sites and the peritoneum in the rats of the normal group. From days 15 to 21, the sensitized rats were intranasally challenged by daily droppings with OVA diluted with sterile physiological saline (50 μL/nostril, 100 mg/mL, 10% OVA). The normal group rats were intranasally challenged by daily droppings with normal saline.

After the OVA‐induced AR model was successfully established, the rats were randomly divided into 5 groups (*n* = 12 per group). In addition to the normal group and model group, the other 3 groups of rats received different frequencies of MSC treatment by intraperitoneal injection: group A (rats treated with MSCs once 7 days prior to AR rat model construction), group B (rats treated with MSCs once a day after AR rat model construction), and group C (rats treated with MSCs weekly for 4 consecutive weeks after AR rat model construction). Approximately 5 × 10^6^ MCSs were suspended in 0.5 mL of saline and intraperitoneally injected into each rat.

### 2.4 Evaluation of nasal symptoms

On the 30th day from the first injection of MSC cells, the rats were stimulated by the dropping of OVA (20 μL/nostril, 50 mg/mL) dissolved in a physiological saline solution into the bilateral nasal cavities, and maintained in the observation cage. Instances of sneezing and nose scratching were counted for 10 minutes after the OVA intranasal stimulation to evaluate the degree of allergic responses. The control rats were processed with saline.

### 2.5 Histological analysis of nasal mucosa

7After the experiment, the rats were sacrificed by drawing out all the blood in the hearts after isoflurane anaesthesia, and the nasal mucosa was separated and fixed in 4% paraformaldehyde at room temperature. After fixation, the tissues were routinely paraffin embedded, sectioned at 4 μm thickness, and stained with haematoxylin and eosin to examine eosinophils, lesions, and swelling of the nasal mucosa.

### 2.6 Serum and plasma

Abdominal aorta blood samples (at least 10 mL) were collected in vacutainer tubes under sterile conditions from the normal, model, and treatment groups of rats. Serum and plasma were obtained following rapid centrifugation and stored at −80°C.

### 2.7 Cytokine examinations

Serum IL‐4, IL‐17, tumour necrosis factor alpha (TNF‐α), IFN‐γ, IgE, and histamine expression levels were measured by enzyme‐linked immunosorbent assay (ELISA; IL‐4 and IFN‐γ ELISA kits, MUTI SCIENCE, China; IgE and histamine ELISA kits, eBioscience, USA), according to the manufacturer's instructions. Absorbance was measured at 450 nm by a microplate reader (Bio‐RAD Model 680, USA). Each measurement was repeated in triplicate, and the mean value was recorded (pg/mL).

### 2.8 Statistical analysis

Statistical analyses for the experimental studies were carried out by using Student *t* tests. The values are shown as the mean ± standard error from at least 3 experiments. Statistical significance was set at a *P* value of less than .05.

## 3 RESULTS

### 3.1 Mesenchymal stromal cells inhibit the symptoms of sneezing and nose rubbing motions in allergic rhinitis rats

**Figure 1:**
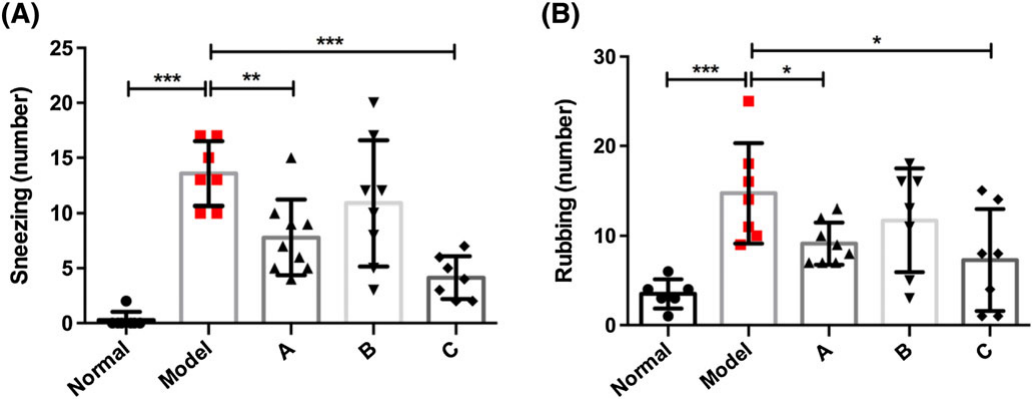
(A) Changes in sneezing number in the normal, model, and different mesenchymal stromal cell (MSC) treatment groups of rats. (B) Changes in nose scratching number in the normal, model, and different MSC treatment groups of rats. *P < .05, **P < .01, and ***P < .001 compared with the model group (normal: untreated wild‐type rats; model: ovalbumin (OVA)‐induced acute allergic rhinitis rat model; (A) rats treated with MCSs once a week before allergic rhinitis (AR) rat model construction; (B) rats treated with MCSs once a day after AR rat model construction; (C) rats treated with MCSs weekly for 4 consecutive weeks after AR rat model construction)

To investigate the role of MSCs in AR, we used an AR rat model and administered OVA with aluminium hydroxide gel for 30 days. We measured the number of sneezing and nose rubbing motions in 10 minutes after challenging the rats with OVA via nasal inhalation for 30 minutes after the rats were treated with MSCs by using different therapeutic strategies. The results showed that the rats in the model group sneezed (*P* < .001) and rubbed (*P* < .001) their noses significantly more frequently than those in the normal group. Interestingly, the sneezing and nasal rubbing numbers were lower in the rats treated with multiple dosages of MCSs from the commencement of OVA administration (Figure 1A, group A compared with group model, *P* = .001); however, a one‐time treatment with MCSs before OVA administration had no significant effect (Figure 1A, group B compared with group A, *P* = .141). Interestingly, an improved strategy with MCSs administered weekly for 4 consecutive weeks after the commencement of OVA administration demonstrated a much better therapeutic effect on the inhibition of sneezing (Figure 1A, group C compared with group model, *P* = .0001). Simultaneously, we observed that the rubbing numbers of the rats showed a similar change after treatments with different therapeutic strategies. In particular, the improved therapeutic strategy (group C) had the best effect on the inhibition of nasal rubbing among all the treatment groups (Figure 1B, group C compared with group model). This result suggests that MSCs have a therapeutic effect on acute AR rats.

### 3.2 The effect of mesenchymal stromal cells on the histology changes of the nasal mucosa in allergic rhinitis rats

**Figure 2:**
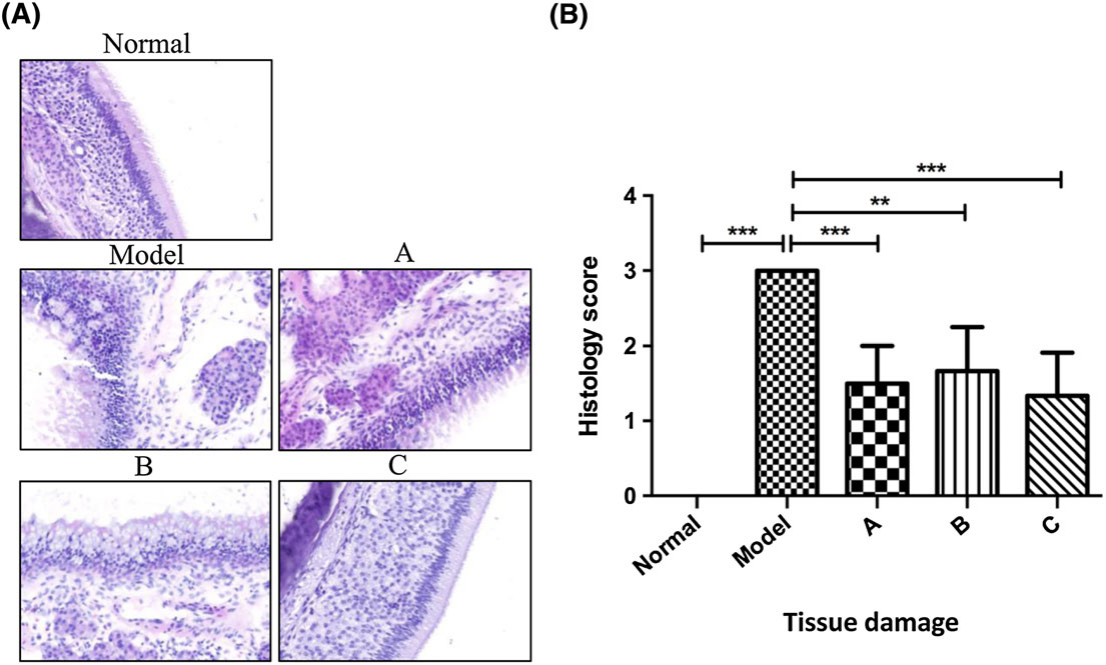
(A) Histological features of the nasal mucosa in the normal, model, and different mesenchymal stromal cell (MSC) treatment groups of rats (haematoxylin and eosin staining; magnification, ×100). (B) Quantification of the severity of the tissue damage using the standard histology scoring system. *P < .05, **P < .01, and ***P < .001

The effect of MSCs on the histology changes in nasal mucosa was evaluated by haematoxylin and eosin staining in our study. The results showed that the administration of OVA caused significant changes in the structure of the nasal mucosa, as the epithelial cells lost their positions, mucosa exfoliation occurred, and eosinophils infiltrated the basal stromal layer compared with the normal tissue (Figure 2A, group model compared with group normal). Interestingly, all the treatments with MSCs showed remarkable recovery of the pathological abnormalities (Figure 2, groups A, B, and C compared with group model). In particular, the improved therapeutic strategy with multiple dosages significantly protected the nasal mucosa from the damage of OVA stimulation (Figure 2A, group C vs group model). The nasal mucosa tissue in the rats treated with multiple dosages of MSCs showed almost no differences compared with the normal rats (Figure 2A, group C compared with group normal), suggesting that treatment with MSCs could inhibit the damage of the epithelial tissues caused by the disease. We further quantified the severity of the tissue damage by using the standard histology scoring system. The results showed that the multiple dosage treatment had a better therapeutic effect on the recovery of the pathological damages (Figure 2B, group C compared with group model). All these results indicate that MSCs prevented nasal destruction and multiple treatments enhanced the recovery of destroyed nasal mucosa.

### 3.3 Mesenchymal stromal cells reduce the expression of interleukin 4, tumour necrosis factor alpha, and immunoglobulin E in the serum of allergic rhinitis rats

**Figure 3:**
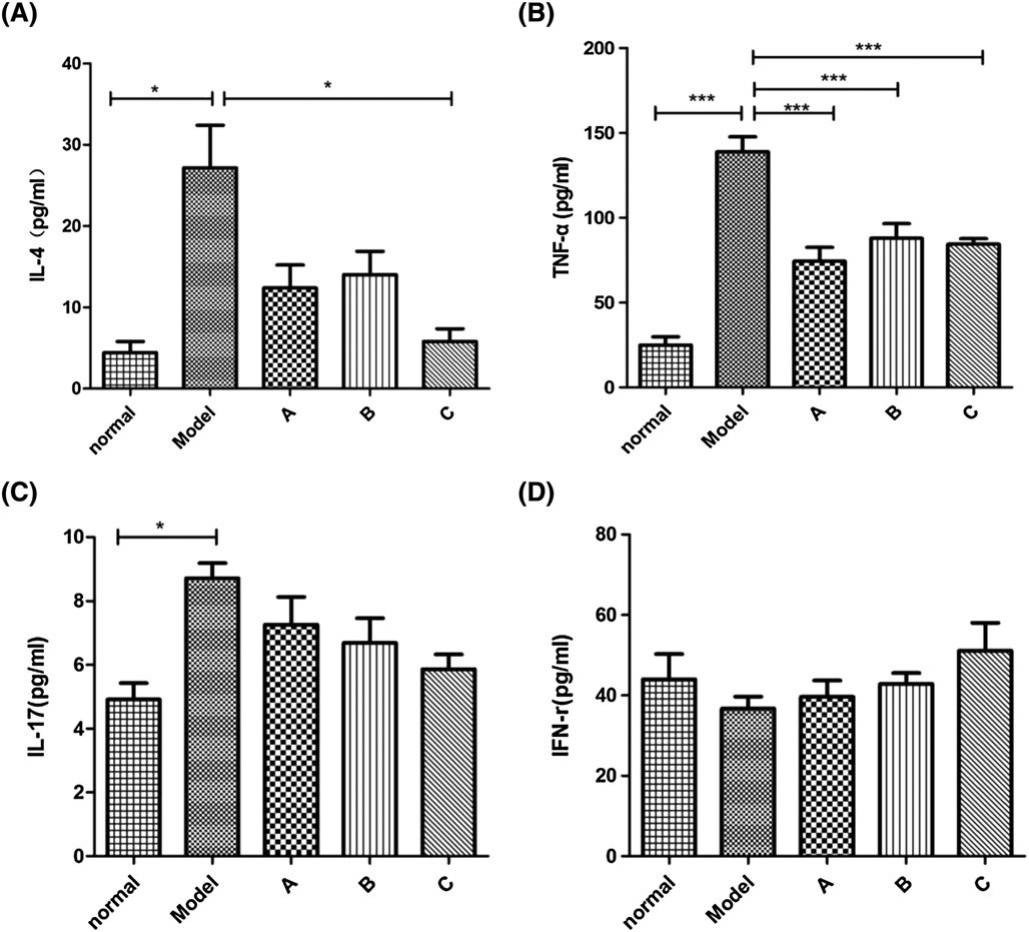
Changes in cytokines in the serum of rats. (A) Mesenchymal stromal cell (MCS) treatment 4 times after the establishment of ovalbumin (OVA)‐induced acute allergic rhinitis in rats reduced the expression of interleukin 4 (IL‐4) in the serum of the rats. (B) Three strategies of MCS treatment all dramatically decreased the expression of tumour necrosis factor alpha (TNF‐α) in the serum of rats. (C and D) Although the expression of IL‐17 and interferon gamma (IFN‐γ) have no statistical significance between the OVA‐induced acute allergic rhinitis rats and the MCS treatment groups, the results correspond with the prospective tendency. *P < .05, **P < .01, and ***P < .001

To elucidate the mechanism underlying the therapeutic effect of MSCs on AR, we examined the production of cytokines that mediate the symptoms of the disease. We measured the expression level of IL‐4, IL‐17, TNF‐α, and IFN‐γ, as those have been demonstrated to play a key role in AR.^7^, ^11^, ^12^ The results showed that the level of IL‐4 was dramatically decreased by the multiple dosage treatment (Figure 3A, group C), and the other 2 strategies were also effective (Figure 3A, groups A and B). Furthermore, the level of TNF‐α was decreased significantly after the different treatments with MSCs (Figure 3B). It appeared that all of the treatments had similar significant effects on the inhibition of the production of TNF‐α. On the other hand, the levels of IL‐17 and IFN‐γ were not significantly changed after the MCS treatments, although IL‐17 tended to decrease (Figure 3C) and INF‐γ tended to increase slightly (Figure 3D). Together, these results indicate that MSCs play a role in the regulation of the production of inflammatory cytokines, in particular, IL‐4 and TNF‐α.

### 3.4 Mesenchymal stromal cells reduce histamine in the plasma of allergic rhinitis rats

**Figure 4:**
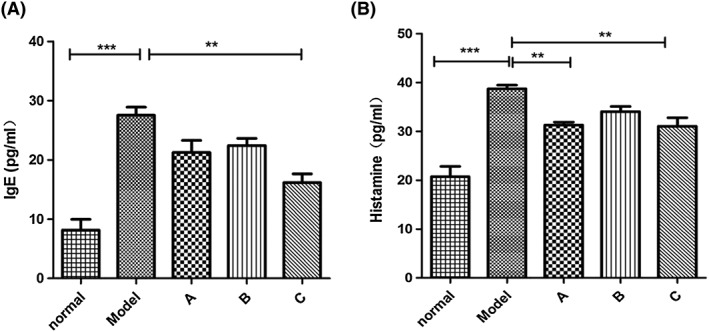
(A) Mesenchymal stromal cell (MCS) treatment 4 times after the establishment of ovalbumin (OVA)‐induced acute allergic rhinitis in rats significantly attenuated the expression of immunoglobulin E (IgE) in the serum of the rats. (B) MCS treatment once before or 4 times after the establishment of OVA‐induced acute allergic rhinitis in rats obviously reduced the expression of histamine in the serum of the rats. *P < .05, **P < .01, and ***P < .001

Antigen‐induced IgE and histamine release from mast cell degranulation are the features of AR.^8^, ^10^ To further examine the therapeutic effect of MSCs on AR, we measured the expression level of IgE in the serum and histamine in the plasma in each group of rats via ELISA. Our results displayed that the multiple dosage treatment significantly decreased the level of IgE (Figure 4A) and inhibited histamine release caused by OVA stimulation in AR rats (Figure 4B). These results suggest that MSCs function in inhibiting mast cells, preventing them from secreting histamine. These results are consistent with the symptom amelioration of acute AR in rats.

## 4 DISCUSSIONS

Allergic rhinitis is a common inflammatory disease worldwide.^1^, ^4^, ^5^ Patients have a well‐defined phenotype, including increased airway resistance, oral breathing difficulty, sleep disturbance, and problems with social activities.^2^, ^6^ Current chemical drugs towards AR are limited to antihistamines, antiallergens, antileukotrienes, and intranasal corticosteroids that only alleviate allergic symptoms but fail to regulate the allergic reaction. Occasionally, these drugs have side effects, such as headache, throat irritation, and nasal dryness.^17^, ^18^, ^19^ Thus, the development of more safe and effective therapeutic agents is needed.

Mesenchymal stromal cells have been proven to possess immunomodulatory and antiinflammatory properties.^20^ Currently, human MSCs hold great promise in regenerative medicine for the therapy of human diseases.^14^, ^15^, ^16^ Human MCSs can be acquired from many tissues, such as umbilical cord, bone marrow, and chorionic membrane.^13^ Many studies have focused on the characteristics of MSCs from different sources for cell therapy. Consistently, the chorionic membrane and umbilical cord are considered good options for clinical application because of their characteristics of self‐renewal and differentiation and mainly for their paracrine immunomodulatory and immunosuppressive effects with lower donor‐associated variability.^21^, ^22^ Further, the clinical uses of mesenchymal stem cells are not encumbered by ethical considerations or legal restrictions.^23^

The MSCs used in this study were isolated from human umbilical cord. The successful study of the pathogenesis and pathophysiology is dependent upon the establishment of a successful animal model of AR. Animal models induced by intraperitoneal injections of allergens vary greatly from the natural processes of AR. It has been reported that MSCs mediate their immunomodulatory effects by interacting with adaptive immunity systems; an altered Th1/Th2 balance in allergic diathesis has recently been termed a “procrustean paradigm”.^24^ T helper type 2 are activated by proinflammatory cytokines such as IL‐4 that are produced in the process of AR.^25^ The ability of MSCs to significantly decrease the secretion of IL‐4 is the main reason why the injection of MSCs is effective in AR. Furthermore, macrophages are another key player in AR, and we found that MSCs achieve their antiinflammatory effects by reducing the secretion of TNF‐α.

Here, we investigated the role of umbilical cord‐derived human MSCs in AR. Based on our studies, MSCs present a promising immediate curative effect to the inflammatory reaction in AR rats. Both AR symptoms and AR‐related inflammatory cytokines have been inhibited by the intraperitoneal injection of MSCs. Although the continuous weekly therapy of MSCs for 1 month presents the strongest effects of AR in rats, the specification of therapeutic frequency, the dosage of MSCs, and the potential mechanism still need to be investigated further. Additionally, MSC therapy in AR needs to undergo innovations and improvements to be a more effective and safer treatment option for AR patients in the future.

## CONFLICT OF INTERESTS

The authors declare no conflicts of interest.

## AUTHORS' CONTRIBUTIONS

CL conducted most of the experiments, analysed the results, and wrote the paper. YF, YYW, and YK provided overall experimental instruction and critically read the manuscript. ML, DM, and WZ performed the experiments. HW, YL, SL, FR, and JL analysed the data. YL and ZC engaged in critical discussions with CL. YW conceived the idea for the project and engaged in discussions.
